# Measuring Patient Experience and Patient Satisfaction—How Are We Doing It and Why Does It Matter? A Comparison of European and U.S. American Approaches

**DOI:** 10.3390/healthcare11060797

**Published:** 2023-03-08

**Authors:** Anna Lena Friedel, Sonja Siegel, Cedric Fabian Kirstein, Monja Gerigk, Ulrike Bingel, Anke Diehl, Oliver Steidle, Steffen Haupeltshofer, Bernhard Andermahr, Witold Chmielewski, Ilonka Kreitschmann-Andermahr

**Affiliations:** 1 Department of Neurosurgery and Spine Surgery, Center for Translational Neuro- and Behavioural Sciences, University Hospital Essen, University of Duisburg-Essen, Hufelandstr. 55, 45147 Essen, Germany; 2 Institute for Medical Education, Center for Translational Neuro- and Behavioural Sciences, University Hospital Essen, University of Duisburg-Essen, Hufelandstr. 55, 45147 Essen, Germany; 3 Institute for Patient Experience, University Hospital Essen, University of Duisburg-Essen, Hohlweg 8, 45147 Essen, Germany; 4 Department of Neurology, Center for Translational Neuro- and Behavioural Sciences, University Hospital Essen, University of Duisburg-Essen, Hufelandstr. 55, 45147 Essen, Germany; 5 Digital Transformation Unit, University Hospital Essen, University of Duisburg-Essen, Hufelandstr. 55, 45147 Essen, Germany; 6 Clinical Quality and Risk Management, University Hospital Essen, University of Duisburg-Essen, Hufelandstr. 55, 45147 Essen, Germany

**Keywords:** patient experience, patient satisfaction, survey, social media, sociodemographic characteristics, hospital characteristics, USA, UK, Scandinavia, Germany

## Abstract

(1) *Background:* Patients’ experiences and satisfaction with their treatment are becoming increasingly important in the context of quality assurance, but the measurement of these parameters is accompanied by several disadvantages such as poor cross-country comparability and methodological problems. The aim of this review is to describe and summarize the process of measuring, publishing, and utilizing patient experience and satisfaction data in countries with highly developed healthcare systems in Europe (Germany, Sweden, Finland, Norway, the United Kingdom) and the USA to identify possible approaches for improvement. (2) *Methods:* Articles published between 2000 and 2021 that address the topics described were identified. Furthermore, patient feedback in social media and the influence of sociodemographic and hospital characteristics on patient satisfaction and experience were evaluated. (3) *Results:* The literature reveals that all countries perform well in collecting patient satisfaction and experience data and making them publicly available. However, due to the use of various different questionnaires, comparability of the results is difficult, and consequences drawn from these data remain largely unclear. (4) *Conclusions*: Surveying patient experience and satisfaction with more unified as well as regularly updated questionnaires would be helpful to eliminate some of the described problems. Additionally, social media platforms must be considered as an increasingly important source to expand the range of patient feedback.

## 1. Introduction

Patients’ experiences of their own treatment and their satisfaction increasingly move into the focus as key quality indicators in many countries with highly developed healthcare systems. According to Bull [1,2], patient experience can be defined as “what” happened during an episode of care and “how” it happened from the patient’s perspective, whereas patient satisfaction rather captures the personal expectations and subjective opinions of the received care. Although both concepts are not interchangeable, they are complexly related, having a profound influence on each other and on treatment outcome [3,4]. Healthcare providers and researchers use patient experience and satisfaction scorings for general, indication-based, and disease-specific patient feedback [5] as tools to improve patient-centered healthcare or due to requirements by government or other regulatory authorities to conduct patient surveys on a regular basis. With respect to the evaluation of their impression of health service delivery, patients’ feedback on their treatment has also become an economic factor since reimbursement as well as the reputation of hospitals in some healthcare systems are also dependent on patients’ judgements of their received care [6].

To assess the patient view on received care, the treatment process, and related factors in a standardized way, patient-reported outcome measures (PROM) and patient-reported experience measures (PREM) are commonly used. Whereas PROM usually question specific aspects of treatment outcome by means of questionnaires, e.g., on health-related quality of life, PREM gather information on patient view of their health service experience and thus allow direct feedback to healthcare providers with the intention of improving the system and achieving integrative care [7]. However, there is a huge variety of approaches even in countries with high-quality healthcare, which is partly predefined by the different orientations and mandates of these systems. As a consequence, the already-existing PREM-surveys (e.g., HCAHPS, PPE-15, and PEQ) differ in validity and reliability [2].

In the face of this complex mix of issues, it is the aim of this review to describe and summarize the process of measuring, publishing, and utilizing patient experience and satisfaction data in countries with highly developed healthcare systems in Europe and the USA in order to identify possible leverage points for improving the collection of and consequences drawn from this important source of information.

## 2. Materials and Methods

### Article Search and Selection Strategy

To incorporate the issues raised above, we opted for the preparation of a narrative review using the following approach: We studied healthcare rankings and reports (WHO report 2000 [8,9], KPMG report 2017 [10]; OECD report 2001 and 2019 [11,12]) in order to identify countries with highly developed healthcare systems that have a long history of measuring patient satisfaction on a regular basis but have different health system structures, reimbursement strategies, and access options for patients. These criteria, chosen to cover a wide spectrum of possibilities to implement the issue of patient satisfaction, resulted in the identification of the USA, UK, and Nordic countries (Norway, Sweden, and Finland). Therefore, studies from these countries were primarily selected for evaluation. Additionally, studies from the German healthcare system were included for comparison. Because the Internet has become one of the most important and easily accessible sources of information and feedback for patients, we also explicitly searched for studies reporting the issue of patient experience and patient satisfaction in social media.

The database search was conducted in *PubMed* (*Medline* database primarily), *Google Scholar*, and *Google* in 2020–2021. Scientific articles, health reports, dissertations, and websites published between 2000 and 2021 in the English or German language were screened. Selected articles were also examined for references as an additional source for this review, leading to the inclusion of a few older articles. The following catchphrases were used during the database search: “patient”, “satisfaction”, “questionnaire”, “survey”, “patient experience”, “patient perspective, and “patient satisfaction” combined (“AND”) with “social media”, “hospital characteristics”, “socio-demographic characteristics”, “Germany”, “USA”, “UK”, “Scandinavia”, “Norway”, “Sweden”, or “Finland” and any combination (“AND”, “OR”) of the terms. The governmental healthcare surveys of the mentioned countries were used as additional search parameters for extracting information if available. A more detailed flow chart of the literature search with catchphrases for the different subsections as well as in- and exclusion criteria is provided as a flow chart as Supplementary Materials.

Inclusion and quality assessment were performed by interdisciplinary discussion among the authors of this article.

## 3. Results

The results section starts with an overview on the most frequently used PREMS measuring patient experience and moves on to present data on this topic according to the countries mentioned above. We then provide the reader with an overview of studies on emerging new platforms of patient feedback, such as Internet databases, as well as studies analyzing the impact of sociodemographic and hospital characteristics. Whenever possible, we differentiated between the concept of patient experience and patient satisfaction. If the cited sources did not allow to make this distinction, we used both terms.

### 3.1. Surveys Measuring Patient Experience

In the countries focused on in this review, the most widely used instruments over the past 20 years are the Picker Patient Experience Questionnaire (PPE)-15 [13], the Hospital and Consumer Assessment of Healthcare Providers and Systems (HCAHPS) [14] (USA), the National Health Service Inpatient Survey (NHSIP) [15] (United Kingdom), and the Patient Experience Questionnaire (PEQ) (initially used in Norway) [16,17]. In Scandinavian countries, patient satisfaction is additionally measured with a variety of questionnaires tailored to country-specific healthcare aspects [18,19]. Since the numbers of patients investigated by these surveys are rather small, the instruments used are not described in more detail here. The four most widely used instruments for measuring patient experience of hospital treatment and their dimensions are described below. Their major characteristics are summarized in Table 1.

#### 3.1.1. The Picker Patient Experience Questionnaire (PPE)-15

The PPE, as the first systematic assessment of patient experience, was developed and has been disseminated throughout the USA since 1987 and, since 1998, also in Europe [22]. The original instrument contained 40 items based on a systematic literature review, expert consultations, the conduction of patient focus groups, and in-depth interviews interrogating patient healthcare experiences. The current Picker Adult Inpatient Survey (PPE-15) [13], in use since 2002, is a revised and shortened version of the original PPE questionnaire. It now contains 15 items out of the original 40, querying issues from information and education to continuity and transition of healthcare. The questionnaire is intended to define problematic aspects of patients’ in-hospital stay that patients believe could be improved. Therefore, a dichotomous “problem score” indicating the presence or absence of a healthcare problem is derived from each item and used for statistical analysis. Based on their face validity, the items are grouped into eight dimensions (cf. Table 1) that have emerged as the most salient issues in patients’ experience of hospital care [23].

#### 3.1.2. The Patient Experience Questionnaire (PEQ)

The PEQ was introduced as a new consultation-specific questionnaire of patient experiences in Norway in 2000 [17]. It was developed to improve the quality of care, with a special focus on the doctor–patient relationship in the inpatient setting and for national surveillance purposes [17,24]. The original survey includes 18 items questioning five dimensions that measure the satisfaction of patients during their stay in medical institutions (cf. Table 1). Initiated in 2005, a modified version of the PEQ was developed in Germany in cooperation with two large, national statutory health insurances (AOK [25] and BARMER [26]) and the Bertelsmann Foundation [27] and has been used for the measurement of patient experience there on a regular basis since November 2011 [28,29].

Since the release of the original PEQ, this questionnaire has been used mainly in the Scandinavian countries as a template to develop more specific questionnaires for certain patient groups or healthcare questions [19].

#### 3.1.3. National Health Service Inpatient Survey (NHSIP)

In 2001, the U.K. implemented the systematic measurement of patient experience as an essential part of their healthcare system (the National Health Service, NHS [30]), with the aim to make NHS more patient-centered and responsive to patient feedback [31]. Patient experience with in-hospital treatment has been measured with the NHS inpatient survey (NHSIP) [32] since 2002 [33]. This instrument was derived from the early Picker Adult Inpatient Survey and adapted for use in the NHS based on the outcome of qualitative research measures (focus groups and cognitive interviews with patients) conducted by the Picker Institute Europe [20]. The inpatient survey is supplemented by surveys focusing on a variety of services and patient groups, including, for example, the experiences of children and adolescents or of patients in urgent and emergency care [34].

The current NHSIP consists of eight dimensions with 49 questions and is implemented on a nationwide basis through postal administration [15]. In contrast to other inpatient surveys, the NHSIP questions are reviewed and potentially revised each year to ensure their ongoing importance for patients and therefore for the NHS [35].

#### 3.1.4. The Hospital Consumer Assessment of Healthcare Providers and Systems (HCAHPS)

The HCAHPS survey is the first nationwide, standardized, publicly reported survey of patient perspectives on hospital care in the USA [36]. It was developed by the Agency for Healthcare Research and Quality and the Centers of Medicare and Medicaid Service (CMS) in 2002 in a process involving literature reviews, cognitive interviews, consumer focus groups, and stakeholder input [21,37]. The HCAHPS inpatient survey was rolled out in 2006 on a voluntary basis, and linkage to hospital payment followed in fiscal year 2008 [21]. It currently contains 29 items and 10 dimensions [37], which are described in more detail in Table 1. Originally designed for improving hospital services and quality of care, HCAHPS results have been included in the Hospital Value-based Purchasing (VBP) program since 2012, which rewards acute-care hospitals with incentive payments for the quality of care provided in hospital settings [38].

### 3.2. Measuring Patient Experience in Selected Countries

#### 3.2.1. United States of America (USA)

In the USA, the delivery of healthcare can be regarded as a consumer-driven industry. Most Americans obtain health insurance coverage through employers, private purchase, or government-based programs and the majority of healthcare facilities in the USA are privately owned. For those, high patient satisfaction translates into a competitive advantage in keeping old and attracting new patients. As mentioned above, hospital reimbursement rates are also linked to HCAHPS ratings [21].

The results of the 10 domains of the questionnaire are publicly reported on the HCAHPS website [39] and for individual hospitals on the Hospital Compare website of the CMS [40]. Annual reports describing scores according to geographic region, hospital type, and number of beds are also provided [41]. The HCAHPS reports from recent years indicate that there were no major changes in patient experience over time [39].

Despite the accountability of VBP for HCAHPS scores and the high public visibility of the questionnaire results, only few studies report patient experience of hospital treatments against a scientific background. In 2008, Jha and colleagues reported that a higher ratio of nurses to patient-days led to increased patient satisfaction, whereas other key hospital characteristics, such as profit or academic status, did not [42]. Thirteen years later, Seiler et al. [43] showed that the patients’ overall satisfaction with inpatient care provided by hospitalists and primary care physicians was nearly the same, with no differences among the specific domains of satisfaction, including communication skills, pain control, and physician behavior.

In summary, data collection concerning patient experience and satisfaction is well established in the United States through the predominant use of the HCAHPS. Nevertheless, apart from reimbursement policies, information on the practical implications of the data remains scarce.

#### 3.2.2. United Kingdom (UK)

The public U.K. healthcare system, the NHS, is grounded in the principles of universality, equity, and being cost free at the point of delivery, paid for by central governmental funding to this day [44]. Healthcare providers with good patient experience and satisfaction rankings do not receive monetary incentives, but the surveys are a way of measuring progress, improving healthcare providers, and holding them accountable for their outcomes [45]. All surveys are documented on the NHS website to be reviewed by the public. The data about patient experience and satisfaction obtained by the surveys are used to give a score out of 10 to each hospital (the higher the better), giving more detailed insights about the ranking in each aspect of the questionnaire [46]. The 2021 survey on adult inpatient care found that the majority of patients gave positive reports about the communication with physicians and nurses, felt a sense of confidence and trust in their care, and were treated with dignity and respect. Patients also reported feeling included in conversations and understanding the answers to their questions. Topics in need for improvement included obtained help from the staff when needed, discharge management, and care at home [47]. Furthermore, data about the development of patient experience over a period of 10 years are provided [48].

Additionally, authors of various studies used the provided or collected additional data to perform secondary analyses of specific attributes. For example, Reeves and West analyzed the data of the NHSIPs from 2002 to 2013 in England, comprising 840.077 patients. They found improvement regarding obtaining copies of physician letters, gender-neutral accommodation on the wards, clinicians and general ward hygiene, as well as waiting times upon admission. The authors underline the need for consistency in investigating patient experience and satisfaction to detect changes over a long period since year-to-year changes might be small [49]. Another study questioning a sample of 2249 in-hospital patients in Scotland with the PPE found that important determinants of satisfaction were physical comfort, emotional support, and respect for patient preferences [50].

To summarize, there is a large amount of data collected in the U.K. mainly by the NHSIP regarding patient experience and patient satisfaction. These data are used in particular to ensure and improve quality of care and to provide information to patients without impacting reimbursement.

#### 3.2.3. Norway, Sweden, and Finland

Healthcare systems in most Scandinavian countries (i.e., Finland, Sweden, and Norway) have followed a similar path. They are well established with regard to primary and preventive healthcare and also have highly developed hospitals, with all citizens having equal access to services. They are taxation based and locally administrated but require co-payments by patients for hospital care and medicines [51]. All Nordic countries have a history of measuring patient experience; however, much of this work was (or still is) done at the local level [52].

To improve comparability, the Nordic Patient Experiences Questionnaire (NORPEQ), a diagnostic instrument for assessing patient experiences of hospital care, was developed in the collaborative effort of Nordic countries [53]. The NORPEQ was validated in a Norwegian sample and subsequently translated and validated in other Nordic languages [52]. However, only few studies use the NORPEQ so far. Besides the NORPEQ, many other diagnostic instruments for patient experiences are used, including the PEQ [16], the PPE-15 [13], as well as a wide range of national surveys or instruments adapted to specific patient groups [19].

On the national level, Norway, Finland, and Sweden publish a wide range of epidemiological and aggregated medical data on governmental websites (Helsedata.no [54]; skr.se [55]; socialstyrelsen.se [56]; thl.fi [57]). However, some information is only accessible after registration with the user’s national bank identification number, which means that foreign website visitors are unable to see the information (helsenorge.no). Aggregated patient experience and/or satisfaction data are not easily accessible for international comparisons in all of these countries.

In Norway, the Norwegian Institute of Public Health is responsible for the monitoring of patient experiences. Since 2019, it has conducted an annual national survey on patient experience and satisfaction for the five following years [58]. Reports are available in Norwegian only [59].

Sweden started to annually collect patient experience data in 2001. Information about care providers has been made public in the “Vårdbarometer” (=care barometer) in Swedish [60]. In 2009, a standardized National Patient Survey, the Nationella Patientenkäten, was additionally introduced, collecting and facilitating comparability of patient experience and satisfaction data on the provider level and over time. The results are made available in Swedish and partially in English [61].

In Finland, healthcare providers are obliged to register healthcare visits into a national registry [62]. Apart from the registries, a national patient survey on health and well-being, including questions concerning patient experience, has been conducted since 2017 (FinSote, 2017–2020 [63]; since 2022, Healthy Finland Survey [64]). Full reports on the surveys are publicly available in Finnish [65], and indicator variables can be accessed and compared for regions and over time in English [66].

In Scandinavia, treatment continuity as well as enough time to listen, talk, and explain during the consultation were identified as important factors for patient satisfaction in primary care [67,68]. Waiting-time reduction is considered a key political challenge for health service improvement [69]. Surveys showed a high satisfaction with how patients were received by medical staff in primary care, while communication in areas concerning waiting times, side effects of medications, previous health status, and health-related warning signals were in need of improvement [70].

In summary, Norway, Sweden, and Finland are well positioned to collect data on patient experiences and to publish the results. The use of group-specific instruments and publications in the respective native languages hamper the comparability between different populations and time points.

#### 3.2.4. Germany

The German public healthcare system is based on the principle of solidarity, where all people insured by statutory health insurance (SHI) receive the same ambulatory and hospital care regardless of their financial status. Approximately 87% of all German citizens fall under this statutory healthcare, whereas the rest has private health insurance (PHI) [71]. SHI or PHI has been mandatory for all citizens and permanent residents in Germany since 2009. The split into the two insurance types is unique among countries in the EU [72].

Since 2005, quality management reports, which address both quality of service and quality of care, have been mandatory in Germany and have to be published by all healthcare providers, private or public, to supply patients with information for benchmarking hospitals [73,74]. As part of these reports, patient satisfaction and patient experience are often surveyed as well. A summary of the results is published occasionally and can be accessed free of charge [75]. Recent results can also be found with various online search engines in German (e.g., “Weisse Liste” [76]) specifically designed to find information on hospitals operated, for example, by SH insurers. However, only the latest results can be found here, and longitudinal data on patient experience are not available.

Starting in 2011, various health insurance companies in cooperation with the “Weisse Liste” have been measuring patient satisfaction and experience nationwide with the PEQ [77]. Factors with particular importance for satisfaction from the patient perspective in Germany include interaction with the attending physicians and the nursing staff [78], which had by far the greatest influence on the patients’ willingness to recommend the hospital to others. These results are in line with corresponding results from the USA [42] and the U.K. [47]. Furthermore, the subjective success of the treatment, the kindness of nurses and physicians, general equipment and cleanliness, the admission procedure and food [78,79], a higher staffing per bed, higher process and outcome quality [80], and number of cases per physician [75] were associated with a positive patient experience or higher satisfaction, respectively.

In summary, Germany also performs well in collecting, analyzing, and publishing data on patient experience and patient satisfaction. However, data on overall consequences drawn from these assessments are hard to find.

### 3.3. Online Patient Ratings on Different Platforms

In recent years, patients increasingly use online platforms to express themselves about their medical treatment and hospital stay and to evaluate health service providers as a quick and easy way to voice an opinion. However, this advantage also gave these platforms the reputation of being unreliable and undifferentiated. In fact, they do not have the same methodological quality as validated PREM or PROM but do provide additional and useful information for studying patient experience and satisfaction. Patient rating platforms fall into two types: on the one hand, platforms such as RateMDs [81], Vitals.com [82], Healthgrades [83], and ZocDoc [84] were designed for the purpose of rating and giving feedback explicitly on hospitals and medical providers, whereas access to certain pages is not possible from all parts of the world (e.g., [83]). On the other hand, on social media platforms such as Facebook [85] or other platforms such as Yelp [86], people can express their opinions about a wide variety of topics. Several studies have compared online patient ratings with results from more traditional and established forms of patient ratings or other indicators of quality of care, such as unplanned readmission rates within 30 days after discharge from hospital. One study found that those hospitals with low readmission rates had higher ratings on Facebook [87], while others showed a positive association between the results of the subjective reviews (ratings and comments) on this platform or Yelp and the HCAHPS scores of the respective hospital [88,89,90,91]. Perez and Freedman (2018) found similar results when comparing reviews from Facebook, Yelp, and Google to patient experience measured with HCAHPS. They showed that in 50–60% of cases, the hospitals rated best on crowdsourcing sites were also the best hospitals according to HCAHPS patient experience ratings. In contrast, in about 20% of cases, the hospitals rated best on Facebook, Yelp, and Google were the worst according to patient experiences measured by HCAHPS [92].

In sum, the data suggest that subjective ratings on social media or other platforms can be used as a source of fair to good representation of patient experience and satisfaction. Since traditional surveys cover only a specific subset of aspects of patient satisfaction and experience, online formats can broaden the spectrum of patient feedback. A comparison of domains surveyed by the HCAHPS with the platform Yelp showed that the reviews on Yelp covered 12 additional domains not addressed by the HCAHPS, such as compassion of staff, quality of nursing, facilities, and amenities [91]. The U.K., for example, already enables patients and hospitals to complement their ratings on NHS Choices with narrative feedback, following the example of social media platforms [93]. Moreover, current advances in machine learning with improved automatized analysis of qualitative data—as reviews on online platforms are—are bound to facilitate the analysis of such narrative feedback modalities [94]. However, it must be kept in mind that users of online rating platforms may represent only a specific sociodemographic subgroup of patients and that the survey mode may affect response behavior and be susceptible to manipulation, thus limiting the generalizability of the conclusions obtained of such data [95,96].

### 3.4. Impact of Sociodemogaphic Characteristics

Numerous studies have shown that patients’ sociodemographic characteristics may influence patient experience and patient satisfaction [97,98]. Most of these studies focus on patient satisfaction; however, there are studies that also measure patient experience [78,99]. Further, the reviews concerning this topic often include studies that measure either satisfaction or experience or sometimes studies where this remains unclear [97,98,100].

Patients’ age represents the best-studied influencing sociodemographic factor with the most consistent results [97,100], indicating that older patients tend to be more satisfied with healthcare or showed a higher willingness for recommendation than youngers [75,101,102]. Stahl and colleagues [78] specified that older people were less critical in their interaction with physicians and with regard to the admission process and hospital food than younger ones. In contrast, they were more critical concerning the subjective assessment of the treatment success. Although the majority of study results point in the same direction, some studies found contrary effects. For example, Jaipaul and Rosenthal [103] showed that patient satisfaction increases with age until up to 80 years but then declines. However, the effect of age on patient experience and satisfaction in fact seems to be rather small [78,98].

Besides age, self-perceived health status is, in many studies, a significant positive predictor of patient experience or satisfaction [97,99,101,104,105]. However, the correlations between health status and satisfaction often are very small and only explain a small part of the variance [104,105]. For other sociodemographic characteristics (e.g., gender, education, race, social status, marital status, and religion), only few and ambiguous results are available [79,97,98,99,100,101,102,105].

In summary, the scarce data that exist so far indicate that sociodemographic characteristics do not appear to have a major impact or even a consistent effect on a person’s satisfaction with received healthcare [98,99]. However, sociodemographic characteristics should be taken into account when investigating patient satisfaction or patient experience to control their role as potential predictors or confounders [97].

### 3.5. Impact of Hospital Characteristics

Up to now, only few studies have examined the influence of hospital characteristics on patient experience and patient satisfaction [80]. As in the case of sociodemographic characteristics, both patient satisfaction [104,106] and patient experience [78,107] are measured in the related studies. A relevant factor seems to be the size of the hospital. The vast majority of studies revealed that patients were less satisfied or had a worse experience with a growing number of beds [75,78,80,105]. Furthermore, patients in not-for-profit hospitals were found to have a better experience and a higher willingness for recommendation than patients treated in for-profit hospitals [80,108,109]. Some other characteristics associated positively with patient satisfaction were, for example, specialty focus [110], system membership [108], and academic (versus general) status of the hospital [105], whereas inconsistent associations have been described between patient satisfaction or patient experience and teaching status of the hospital [6,104,107] as well as concerning urban versus rural region [75,106].

Overall, various hospital characteristics potentially influence patient experience and patient satisfaction. However, it is unclear how crucial this effect actually is since some associations were found to be inconsistent [6]. In addition, in some studies, the identified characteristics only explain a small part of the variance [105]. Nevertheless, given the potential influence of these hospital-associated features, care should be taken when using survey data concerning patient experience or patient satisfaction in quality management. The data should be evaluated keeping the potential effects of hospital characteristics in mind [78].

## 4. Discussion

The results of our review indicate that all countries studied have established routines for the measurement and publication of patient experience and satisfaction. The NHS appears to be currently leading the way in this regard not only because of the ongoing adaptation of their measurement tool NHSIP but also because of the integration of traditional quantitative and Internet-based narrative feedback possibilities and their publication on the NHS website. In addition, the Scandinavian countries selected for this overview have professionalized the collection and publication of patient experience and satisfaction data, especially in recent years, while the American approach to patient experience and satisfaction data has remained largely unchanged within the last decade. In Germany, on the other side, patient experience data are collected on a regular basis but only made available to the general population in a simplified form on a non-profit foundation website. Germany is, moreover, the only country of the ones studied here that does not provide annual or longitudinal statistical data on patient satisfaction and/or experience to the public.

Despite the individual countries’ efforts to measure and publish patient experience, consequences drawn from the patient feedback to improve national healthcare systems often remain unclear. The Scandinavian countries investigated here claim to incorporate the results of the PREM and PROM surveys into healthcare reforms, while the USA aims to control improvement processes via financial incentives. In the U.K. and Germany, on the other hand, it is up to healthcare providers and patients to draw conclusions from the data collected. However, despite extensive research, we did not find specific examples of how exactly patient experience and satisfaction measures are used to implement healthcare reforms in any of the countries studied.

Next to discussing how the different countries deal with the issue of patient experience and satisfaction, we also aimed to describe what is known to be important for patients in the individual countries. One factor that seems to be crucial for patient satisfaction across all countries mentioned is communication with physicians and nurses. On this aspect, patients in the U.K. seem to report the most positive experiences about their communication with healthcare professionals.

However, due to various methodological problems, the evaluation of these results on a cross-country level is difficult. In some of the countries studied in this review (i.e., Norway and Germany), survey results are only made available in national languages. The use of different surveys and country-specific modifications of existing questionnaires for subgroups make it difficult to compare findings with other health services or even within the same service over time.

Moreover, the construct validity of the inventories used varies considerably [2]. Many PREM (i.e., HCAHPS, PEQ) were developed years ago and are not updated on a regular basis. Some do not differentiate well between the constructs of patient experience and satisfaction but contain elements of both. However, the distinction between the two constructs is important: The measurement of patient experience is likely to uncover differences in the quality of care provided to individual populations, whereas aspects of patient satisfaction are more likely to be influenced by cultural differences in patient expectations or attitudes.

Further, sociodemographic data, which would allow a more differentiated analysis according to subgroups, are not systematically investigated. Yet, these are important when investigating patient satisfaction or patient experience to control their role as potential predictors or confounders.

Emerging and increasingly important sources of patient experience, such as website-based feedback from social media or discussion fora, are oftentimes viewed as unreliable sources of information. Nevertheless, studies show that these narrative data can be analyzed [94] and then yield important additional information such as the reference to additional and underrepresented dimensions of patient experience (i.e., compassion of staff, quality of nursing, facilities, and amenities) that are not yet captured in the conventional questionnaires [91]. Incorporating such domains into the existing measures could possibly improve the process of evaluating patient experience and satisfaction and provide important feedback to the respective hospitals [88,91,111].

Owing to the complexity of the topic and the sheer overwhelming amount of literature published, the present article was prepared as a narrative review rather than a systematic one. This approach constitutes a certain limitation because it obviously leads to a selection bias. We would, however, like this approach of data presentation to be understood as a starting point for further research and more in-depth study of certain aspects presented here.

## 5. Conclusions

In summary, with regard to patient experience, many countries are exemplary in one aspect or another, but all countries have potential for improvement. The present review presents insights beyond the national borders, aiming to provide a basis for improving the use of patient experience data to benefit healthcare systems. Our results suggest that international efforts to unify methods for measuring patient experience, as already initiated with the development of the NORPEQ, should be advanced further. While the distinction between patient experience and satisfaction is already an essential step towards its systematic assessment, questionnaires should be improved in terms of conceptual clarity and validity to reflect this distinction and to achieve better comparability of results.

Another consequence could be to establish mandatory and standardized recommendations for the collection and publication of patient experience data. Summarized results should be provided transparently for patients’ orientation, whereas more detailed cross-sectional and longitudinal data should be made easily accessible to researchers for national and international comparison.

Last but not least, at present, the consequences of patient experience surveys in the different countries are not easily discernible. We suggest implementing their results into change management structures, which have to be constantly adapted to altering healthcare challenges accounting for, e.g., multicultural social backgrounds and minority and marginalized groups. Planned and realized changes to improve patient care should be made easily accessible to the public. In our opinion, such an approach could bring about a vibrant culture of change in healthcare co-designed by patients and healthcare providers and give more comprehensive answers to the question of “how are we doing it and why does it matter”. We hope that the present review can be regarded, in this respect, as a starting point for further research and practical implementations.

## Figures and Tables

**Table 1:** Selected instruments capturing patient experience.

Instrument	Picker Patient Experience Questionnaire (PPE)-15	Patient Experience Questionnaire (PEQ)	National Health Service Inpatient Survey (NHSIP)	Hospital Consumer Assessment of Healthcare and Systems (HCAHPS)
**Reference**	Jenkinson et al., 2002 [13]	Steine et al., 2001 [17]	Reeves et al., 2002 [20]	Giordano et al., 2009 [21]
**Country of development**	USA	Norway	U.K.	USA
**First version**	1987	2000	2002	2006
**Latest version**	2002	2000	2021	2019
**Items**	15	18	49	29
**Dimensions**	Information and education	Communication	Admission to hospital	Communication with doctors
	Coordination of care	Emotions	Hospital and ward	Communication with nurses
	Physical comfort	Short-term outcome	Doctors	Responsiveness of hospital staff
	Emotional support	Barriers	Nurses	Communication about medicines
	Respect for patient preferences	Relations with auxiliary stuff	Care and treatment	Discharge information
	Involvement of family and friends		Operations and procedures	Care transition
	Continuity and transition		Leaving hospital	Cleanliness of the hospital environment
	Overall impression		Overall experience	Quietness of the hospital environment
				Overall rating of hospital
				Recommendation of hospital

## Data Availability

Not applicable.

## Supplementary Materials

The following supporting information can be downloaded at: https://www.mdpi.com/article/10.3390/healthcare11060797/s1, Figure S1: Detailed flow chart of literature search, in- and exclusion criteria.
