# Supplementary figures and images for: Measuring Patient Experience and Patient Satisfaction—How Are We Doing It and Why Does It Matter? A Comparison of European and U.S. American Approaches
Source: Healthcare (Basel). 2023 Mar 8;11(6):797. doi: 10.3390/healthcare11060797 (PMC10048416; doi:10.3390/healthcare11060797)

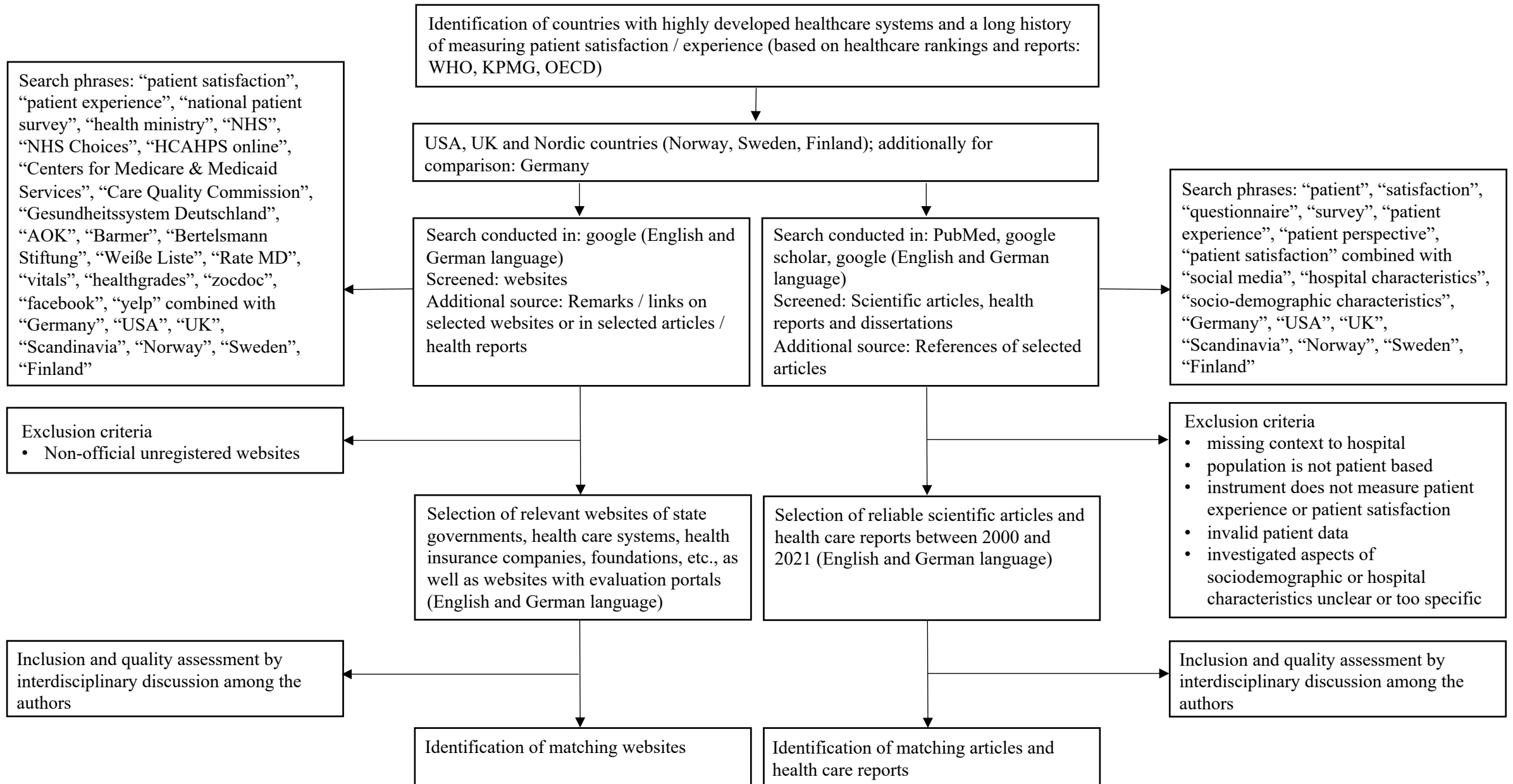

Supplement: Supplementary file 1 [file healthcare-11-00797-s001.zip › healthcare-2081401-supplementary.pdf]
